# Faster N Release, but Not C Loss, From Leaf Litter of Invasives Compared to Native Species in Mediterranean Ecosystems

**DOI:** 10.3389/fpls.2018.00534

**Published:** 2018-04-24

**Authors:** Guido Incerti, Fabrizio Cartenì, Gaspare Cesarano, Tushar C. Sarker, Ahmed M. Abd El-Gawad, Rosaria D'Ascoli, Giuliano Bonanomi, Francesco Giannino

**Affiliations:** ^1^Department of Agri-Food, Animal and Environmental Sciences, University of Udine, Udine, Italy; ^2^Department of Agricultural Sciences, University of Naples Federico II, Portici, Italy; ^3^Department of Botany, Faculty of Sciences, Mansoura University, Al Dakahllia, Egypt; ^4^Dipartimento di Scienze Tecnologie Ambientali Biologiche e Farmaceutiche, Università degli Studi della Campania Luigi Vanvitelli, Caserta, Italy

**Keywords:** litter decomposition, plant invasion, exotic plant species, C/N ratio, lignin/N ratio, ^13^C CPMAS NMR, mass ratio theory, whole-community approach

## Abstract

Plant invasions can have relevant impacts on biogeochemical cycles, whose extent, in Mediterranean ecosystems, have not yet been systematically assessed comparing litter carbon (C) and nitrogen (N) dynamics between invasive plants and native communities. We carried out a 1-year litterbag experiment in 4 different plant communities (grassland, sand dune, riparian and mixed forests) on 8 invasives and 24 autochthonous plant species, used as control. Plant litter was characterized for mass loss, N release, proximate lignin and litter chemistry by ^13^C CPMAS NMR. Native and invasive species showed significant differences in litter chemical traits, with invaders generally showing higher N concentration and lower lignin/N ratio. Mass loss data revealed no consistent differences between native and invasive species, although some woody and vine invaders showed exceptionally high decomposition rate. In contrast, N release rate from litter was faster for invasive plants compared to native species. N concentration, lignin content and relative abundance of methoxyl and N-alkyl C region from ^13^C CPMAS NMR spectra were the parameters that better explained mass loss and N mineralization rates. Our findings demonstrate that during litter decomposition invasive species litter has no different decomposition rates but greater N release rate compared to natives. Accordingly, invasives are expected to affect N cycle in Mediterranean plant communities, possibly promoting a shift of plant assemblages.

## Introduction

Ecosystem invasion by non-native exotic plant species is a global phenomenon with important consequences at ecologic, economic, and social level (Pimentel et al., 2000), and is considered among the most serious threats to biodiversity in terrestrial ecosystems (Hulme et al., 2009). Invasive plant species can alter ecosystem functions by changing fire (D'Antonio and Vitousek, 1992)and hydrological regimes (Le Maitre et al., 1996), as well as carbon (C) and nitrogen (N) cycles (Ehrenfeld, 2003) and stocks, by affecting several processes including N fixation (Vitousek and Walker, 1989), soil N mineralization (Hawkes et al., 2005), plant nutrient uptake (Windham and Ehrenfeld, 2003), and nutrient transfer to soil trough litterfall (Lindsay and French, 2005).

Litter decomposition is a critical process in terrestrial ecosystems, controlling C and nutrient transfer to soil and, consequently, community structure and functions (Facelli and Pickett, 1991; Attiwill and Adams, 1993). The process can be leaded by both biotic (Hättenschwiler et al., 2005) and abiotic factors (Austin and Vivanco, 2006). The rate of litter decomposition is affected by climatic variables, litter quality, and soil biota (Aerts, 1997; Bradford et al., 2016), whose relative importance can change with spatial scale (Liski et al., 2003). In Mediterranean ecosystems, the persistence of summer drought conditions can be critical for litter decomposition, with rainfall sporadically wetting the litter layer in turns subject to drying much faster than the underground soil (Criquet et al., 2004; Incerti et al., 2011). On the other hand, milder and wetter conditions in spring and fall enhance microbial metabolism, with temporarily high litter decay rates (Coûteaux et al., 1995). Another study in Mediterranean environments showed that CO_2_ efflux pulses observed after precipitation can be primarily ascribed to heterotrophic respiration, likely caused by the degradation of easily decomposable substrates accumulated in the soil during the preceding dry periods (Inglima et al., 2009). Moreover, leaf adaptations to limit water loss during summer drought, correspond to high content of structural compounds, with consequent high leaf mass, and low N content per unitary area (Kazakou et al., 2009), which can slow down litter decomposition (Cornwell et al., 2008).

In general, early studies indicated that litter of invasive species usually decomposes at a faster rate compared to that of native plants (Vitousek et al., 1987; Grout et al., 1997; Ehrenfeld et al., 2001). In the first review concerning the effect of invasive species on C and N cycling, Ehrenfeld (2003) reported that litter of exotic plants decomposed more rapidly than that of natives in 10 cases out of 14. Few years later, Liao et al. (2008), based on 94 experimental studies, strongly confirmed the earlier results, reporting that litter decomposition rate in invaded ecosystems was on average 117% higher than in native areas. Such general trends have been related to specific traits of the exotic plants positively associated with rapid growth and fast decomposition, such as N fixation capability, high leaf N content, low specific leaf area, and short life-span. However, the recent meta-analysis by Castro-Díez et al. (2014) partially challenged early evidence showing that plant invasion increased N pools, litter inputs and soil mineralization while, surprisingly, the impact on litter decomposition was not significant, despite C/N ratio was lower in litter of invasive compared to native plants (Castro-Díez et al., 2014).

Based on previous evidence on their relationships with litter decay rate (Meentemeyer, 1978; Melillo et al, 1982; Taylor et al., 1989), C/N ratio, lignin content and lignin/N ratio have been largely used in meta-analyses to compare litter quality of invasive and native species (Liao et al., 2008; Castro-Díez et al., 2014). However, their use to predict decomposition dynamics have been recently criticized. Hättenschwiler et al. (2011) showed how both C/N and lignin/N ratios were poor predictors of litter decomposition rates of several tree species of lowland Amazonian forest. More recently, Bonanomi et al. (2013) provided evidence that C/N and lignin/N ratios, while being good descriptors of litter quality of undecomposed materials, were unable to predict mass loss rate after the early decomposition stage. In the same study, an approach based on ^13^C-CPMAS NMR analysis led to descriptors of litter chemistry more predictive of decay rates throughout the incubation period. The possibility of using ^13^C-CPMAS NMR data to operationally define litter chemical quality is well established (Almendros et al., 2000; Kögel-Knabner, 2002; Mathers et al., 2007; Preston et al., 2009) but, so far, no systematic comparison between invasive and native co-occurring plants has been reported. Another major recognized drawback of previous decomposition studies about invasive species has been related to case-selection sampling bias (Hulme et al., 2013), because only one or few native species were compared with exotic plants (Liao et al., 2008; Castro-Díez et al., 2014) possibly affecting, when not amplifying, the estimate of decay rate differences between invasive and native plant litters. Comparing phylogenetically related native and exotic species could allow to overcome such sample selection-bias, as showed by Ashton et al. (2005) in a comparative analysis of mass loss and nutrient release from litter in four plant species pairs, each made of two phylogenetically related either invasive or native species. However, this approach is only applicable under particular circumstances, where co-occurring native and exotic plants have certain phylogenetic relationships. An alternative approach is to use all the species present in the community, each weighted by its relative abundance, as the native control (Castro-Díez et al., 2014). Although the application of such whole-community approach would be laborious, time-consuming and resource-demanding, especially in species-rich ecosystems, disregarding species abundance in the community might lead to improper interpretation of invasive species impact at ecosystem level (Furey et al., 2014). A pragmatic compromise, based on the mass ratio theory (Grime, 1998), would be to use as native control all the species that reach a certain threshold of abundance. To the best of our knowledge, this approach was not yet applied in a comparative analysis of exotic and native species litter decomposition.

In this work, we assess differences in C and N dynamics during litter decomposition of invasive and native species, by a litterbag experiment (Berg and McClaugherty, 2014) based on litters from 32 plant species (24 native and 8 invasive species) in four types of plant community (grassland, scrubland, riparian forest and mixed deciduous-evergreen woodland) in the Mediterranean region. In each community we compared litter mass loss and N release of exotic species to that of all co-occurring native plants belonging to the same growth form (i.e., herbs, vines, woody shrubs, and trees). The selected communities included plant species spanning over a wide range of litter chemical quality. To clarify the role of litter chemistry on the decomposition rate of exotic and native species we combined undecomposed plant litter characterization by ^13^C- CPMAS NMR in solid state (Kögel-Knabner, 2002), with classic elemental and proximate (cellulose and lignin) analyses (Gessner, 2005). The specific hypotheses of this study were:

1. Does litter of exotic and native species show consistent differences in C chemical quality?
2. Does litter of invasive plants decompose and release N at a faster rate than that of native species across different ecosystems?
3. Are C and N mineralization rates associated to specific chemical traits diagnostically detectable by ^13^C-CPMAS NMR?

## Materials and methods

### Litter collection and decomposition experiment

A multi-sites, 1-year long leaf litter decomposition experiment was carried out in Campania Region (Southern Italy). For this purpose, four incubation sites, representing the major ecosystem types within the Mediterranean biome, were randomly selected within the following plant communities: semi-natural grassland, sand dune maquis, mixed Mediterranean woodland, and riparian forest dominated by deciduous trees. All incubation sites are still dominated by native species but in the past were subject to a progressive invasion by exotic species. Details on site location, climate, litter layer, plant community and native and invasive species list are reported in Table 1 and Figure S1. Species relative abundance, growth form and N-fixation capability are in Table S1.

**Table 1:** Main characteristics of the four incubation sites: location, climatic data (mean annual temperature; total annual rainfall), plant community type, litter layer thickness, and plant species and growth form.

**Study site**	**Latitude and Longitude**	**Altitude (m a.s.l.)**	**Community type**	**Temperature (°C) and rainfall (mm)**	**Litter layer (cm)**	**Species list**
Portici	40°48′ N 14°20′ E	80	Grassland	17.3–1,195	4.0	*Arum italicum* (perennial forb) *Asphodelus ramosus* (perennial forb) *Dactylis glomerata* (perennial grass) *Festuca drymeja* (perennial grass) Oxalis pes-caprae^*^ (perennial forb) *Plantago lanceolata* (perennial forb) *Taraxacum officinale* (perennial forb) *Trifolium pretense*^†^ (perennial forb)
Cicerale	40°19′ N 15°07′ E	186	Riparian forest	16.9–1,328	3.3	Amorpha fruticosa^*^^†^ (deciduous tree) *Populus nigra* (deciduous tree) *Salix alba* (deciduous tree)
Paestum	40°25′ N 14°59′ E	8	Sand dune maquis	17.5–1,490	5.8	Acacia longifolia^*^^†^ (evergreen shrub) Eucalyptus camaldulensis^*^ (evergreen tree) *Juniperus phoenicea* (evergreen shrub) *Mirtus communis* (evergreen shrub) *Phillyrea latifolia* (evergreen shrub) *Pinus halepensis* (evergreen tree) *Pistacia lentiscus* (evergreen shrub)
Portici	40°48′ N 14°20′ E	80	Mixed forest	17.3–1,195	2.3	Trees Ailanthus altissima^*^(deciduous tree) *Alnus neapolitana*^†^ (deciduous tree) *Arbutus unedo* (evergreen tree) Broussonetia papyrifera^*^(deciduous tree) *Celtis australis* (deciduous tree) *Fraxinus ornus* (deciduous tree) *Olmus campestris* (deciduous tree) *Quercus ilex* (evergreen tree) *Quercus pubescens* (deciduous tree) Robinia pseudoacacia^*^ (deciduous tree) Vines *Clematis vitalba* (vine) *Hedera helix* (vine) Ipomea purpurea^*^ (vine) *Rubus hulmifolius* (vine)

*Invasive species are marked by an asterisk, while N-fixing species are marked by^†^*.

A total of 32 plant species, including 24 native and 8 exotic species were used in the decomposition experiment. Considering the focus of this work, all selected exotic species were chosen among the invasives as categorized by Galasso et al. (2018). Eight species were selected from the grassland ecosystem, 7 from the sand dune maquis, 14 from the mixed forest and 3 from the riparian forest (Table 1). The rationale underlying species selection was to compare invasive species to the whole native co-occurring communities. Thus, for each plant community, leaf litter from invasive species was compared to all the co-occurring native species within the same growth form (i.e., invasive herbs vs. native herbs, invasive vine vs. native vine, and invasive tree vs native tree), while all the native plants reaching a minimum abundance threshold of 3% (Table S1) were used as the control. The choice of such threshold value is indeed subjective. However, considering the mass ratio theory (Grime, 1998), we are confident that in this way all native plants having a relevant impact on C and N cycle at ecosystem level were included in the study.

At each incubation site, leaf litter was collected by net traps during the period of maximum leaf fall from N > 20 randomly selected individuals of each selected species. Freshly abscissed leaves were dried in a ventilated chamber at 30°C until constant weight was reached and then stored at room temperature. In September 2012, terylene litterbags (25 × 25 cm^2^, mesh size 2 mm) were filled with 5 g of dry litter and incubated at 10 different randomly-selected locations at each incubation site on the litter layer, by using metal pegs. A total of 1,280 monospecific litterbags (32 species × 4 sampling dates × 10 replicates) were collected in time, after 30, 90, 180, and 360 days of decomposition in field conditions. Bags were dried in the laboratory (30°C until reaching constant weight) and the remaining material weighed afterwards.

### Litter chemical analysis

Initial materials were characterized for total C and N content by flash combustion of microsamples (5 mg of litter) by an CN-soil elemental analyzer (Flash EA2000 Thermo). Proximate cellulose and lignin content were quantified as acid hydrolysable fraction and acid unhydrolysable materials, respectively (Gessner, 2005). Total C and N content of decomposed materials were also assessed after 90 and 360 days of decomposition. In the case of total N, data were expressed as percent variation (with sign changed) with respect to undecomposed material with value fixed to 0%. Overall, this metric indicates the combined result of N transfer from soil to organic material and mass loss. During decomposition, a value higher and lower than 0% indicated a decrease and an increase of total N content, and then was used as indirect evidence of N release and immobilization, respectively.

Undecomposed materials were characterized by^13^C cross-polarization magic angle spinning (CPMAS) nuclear magnetic resonance (NMR) (Kögel-Knabner, 2002) obtained in solid state and under the same conditions, thus allowing a comparative analysis of the resulting spectra. The spectrometer used was a Bruker AV-300 equipped with a 4 mm wide-bore MAS probe (for further details see (Bonanomi et al., 2013)). Spectral regions and corresponding C types have been identified as reported by Bonanomi et al. (2013): 0–45 ppm = alkyl C (characteristic of lipid waxes, cutins and microbial products); 46–60 ppm = methoxyl and N-alkyl C (characteristic of amino acids and lignin components); 61–90 ppm = O-alkyl C (characteristic of carbohydrates and polysaccharides); 91–110 ppm = di-O-alkyl C (anomeric C1 of celluloses, tannin and lignin components); 111–140 ppm = H- and C- substituted aromatic C (mainly associated with polyphenols, lignin and tannin components); 141–160 ppm O-substituted aromatic C (phenolic and O-aryl C, characteristic of phenols, lignin and tannin components); 161–190 ppm carboxyl C (characteristic of organic acids, amides, esters).

### Data analysis

The effects of species and incubation time on litter mass loss and net N release were tested by two-way ANOVA within each plant community. To test whether invasive plant species produced litter of different quality, we analyzed initial chemistry of plant residues using one-way ANOVA within each plant community. Significant between-groups pairwise differences were tested using Duncan's multiple range *post-hoc* test. Nativity/invasivity fixed effects on litter decay rate and net N release were tested by mixed effect models, with random effects of phylogenetic group (i.e., family) and site, and decomposition time as a continuous covariate. The exponential decay constant (*k*) has been calculated following Berg and McClaugherty (2014) by applying the Olson (1963) model. The model equation was M_t_ = M_0_·^−kt^, where M_0_ is the initial litter mass, M_t_ is the litter mass at time t, and k is the decay rate parameter. Both univariate and multivariate statistics were used to investigate the relationship between the litter decay rate (*k*) or the net N release and the chemical parameters of litter (i.e., N, cellulose, and lignin content, C/N ratio, lignin/N ratio, and data from ^13^C-CPMAS NMR spectra). Simple correlation was extensively tested between litter decay rate or N release and each parameter, including the ^13^C-CPMAS NMR spectral signals. Using a multivariate approach, a principal component analysis (PCA) was performed and, following Legendre and Legendre (1998), litter decay rate, net N release, and dummy variables for plant communities were also plotted as loading vectors on the bi-dimensional PCA space even if not used to compute the eigenvalues of the same ordination space. Statistical analyses were carried out using Statistica 10.0 (StatSoft, Tulsa, OK, USA).

## Results

### Native and invasive plant litter chemistry in different communities

Leaf litter chemistry largely varied among species within each plant community (Table 2). In the grassland, the invasive *Oxalis pes-caprae* had the highest lignin/N ratio and a relatively low N content coupled with a high relative content of *O*-alkyl C (Table 2). Expectedly, most of the grassland species showed lower lignin and higher N content compared to the plants from other communities, as resulting from both elemental, proximate and ^13^C CPMAS NMR analyses (Table 2).

**Table 2:** Chemical traits of 32 plant species litters in four invaded plant communities and decay rate fitting results.

	**Elemental and proximate parameters**					^**13**^**C-CPMAS NMR-derived parameters**							**Decay rate fitting**		
**Ecosystem**	**Cellulose (%)**	**Lignin (%)**	**N (%)**	**C/N**	**Lignin/N**	**Carboxylic C – 161–190 ppm**	**O-substituted aromatic C– 141–160 ppm**	**H-C-substituted aromatic C – 111–140 ppm**	**di-*O*-alkyl C – 91–110 ppm**	***O*-alkyl C – 61–90 ppm**	**Methoxyl C – 46–60 ppm**	**Alkyl C – 0–45 ppm**	**k (d^−1^)**	***R*^2^**	***P***
**GRASSLAND**															
*Arum*	20.0c	1.5c	4.7a	10.7e	0.3f	9.2a	0.8e	4.4d	8.3c	42.9c	9.8a	24.5a	0.013	0.73	0.035
*Asphodelus*	30.8a	3.2c	1.6c	31.6b	2.0d	6.8b	1.4d	4.7d	10.8b	51.7b	7.1b	17.4b	0.013	0.94	0.001
*Dactylis*	18.1c	12.9a	2.1b	24.2c	6.2b	5.0c	2.2c	6.5c	11.6b	51.8b	7.3b	15.6b	0.012	0.87	0.006
*Festuca*	22.6c	8.0b	1.1d	43.6a	7.0b	4.4c	4.2a	12.3a	17.5a	61.8a	0.3c	0.2c	0.002	0.75	0.031
Oxalis^*^	25.1b	13.1a	1.6c	30.2b	9.0a	5.8c	1.8d	4.5d	11.8b	51.6b	6.8b	17.6b	0.009	0.86	0.008
*Plantago*	22.8c	11.2a	1.8c	27.5bc	6.2b	7.9ab	3.3b	8.7b	10.6b	46.7bc	7.1b	15.6b	0.013	0.74	0.032
*Taraxacum*	21.7c	2.9c	2.6a	19.1d	1.1e	9.4a	2.6c	6.7c	9.1bc	41.4c	9.1a	21.7a	0.013	0.70	0.047
*Trifolium*	15.4d	10.1a	4.9a	10.3e	1.7d	9.5a	4.9a	9.6b	9.3b	33.8d	10.6a	22.2a	0.013	0.70	0.046
NC	20.9	9.0	2.3	24.8	4.4	6.5	2.6	7.1	11.3	49.1	7.2	16.2	0.011	0.77	0.028
**RIPARIAN FOREST**															
Amorpha^*^	12.9b	17.2b	4.1a	12.1c	4.2c	9.0a	5.3a	11.1a	8.7b	34.2c	8.5a	23.0a	0.003	0.88	0.005
*Populus*	22.5a	12.5c	1.8c	27.9a	7.0b	6.8b	5.3a	11.0a	12.3a	42.8a	5.8b	16.1b	0.004	0.78	0.021
*Salix*	19.7a	24.4a	2.7b	18.3b	8.9a	4.7c	3.7b	8.3b	9.6b	39.5b	8.4a	25.8a	0.002	0.78	0.022
NC	22.1	14.1	1.9	26.6	7.3	6.5	5.1	10.6	11.9	42.3	6.2	17.4	0.003	0.78	0.022
**SAND DUNE MAQUIS**															
Acacia^*^	17.2b	29.9a	2.5a	20.1e	12.1c	7.6a	6.2b	9.1b	9.6b	33.9b	8.1a	25.5b	0.001	0.83	0.013
Eucalyptus^*^	12.3c	11.7d	1.6b	30.5c	7.2d	7.3a	4.3c	9.2b	7.4b	30.6b	8.0a	33.2a	0.004	0.81	0.015
*Juniperus*	13.0c	23.8b	0.7d	69.3a	33.0a	4.4b	4.8c	8.9b	9.7b	38.4ab	6.2b	27.5b	0.002	0.74	0.032
*Mirtus*	15.8b	10.6d	1.2c	41.1b	8.7d	6.2ab	5.7bc	8.9b	11.4a	42.4a	6.2b	19.2c	0.004	0.95	0.001
*Phillyrea*	10.1d	15.9c	1.8b	27.6d	8.8d	5.6b	3.6c	9.6b	8.9b	39.1ab	8.3a	24.9b	0.002	0.91	0.003
*Pinus*	23.7a	30.2a	1.4c	35.6c	21.6b	4.1b	4.9c	8.7b	11.3a	43.6a	6.6b	20.8c	0.003	0.83	0.013
*Pistacia*	17.9b	23.9b	1.1c	45.4b	21.7b	5.3b	9.3a	12.1a	12.6a	34.7b	4.5c	21.5c	0.002	0.86	0.008
NC	16.6b	21.0b	1.2c	44.9a	19.2b	5.2	6.4	10.0	11.2	38.9	5.9	22.3	0.003	0.86	0.011
**MIXED FOREST TREES**															
Ailanthus^*^	13.4c	13.8d	3.0a	16.6e	4.6d	9.6a	3.7d	8.6c	7.5c	37.4c	8.8a	24.5a	0.013	0.73	0.035
*Alnus*	18.7ab	13.1d	2.9a	16.6e	4.5d	8.7ab	4.0c	10.8b	7.9c	34.9c	8.4a	24.4a	0.009	0.99	<0.001
*Arbutus*	13.0*c*	26.8a	1.3d	39.2a	21.0a	6.9c	7.4a	11.7a	11.1a	37.5c	5.6b	19.9bc	0.001	0.77	0.026
Brussonetia^*^	17.1a	3.3f	2.2b	23.1c	0.8e	6.8c	2.8d	7.8c	10.3ab	44.7a	8.7a	18.9bc	0.013	0.83	0.012
*Celtis*	21.7a	6.5e	2.1c	23.6c	3.1d	9.1a	2.3d	7.5c	9.0b	40.3b	8.8a	23.0a	0.009	0.93	0.001
*Fraxinus*	15.4c	10.0d	2.0c	24.6c	4.9d	5.7c	2.9d	7.8c	10.7a	46.3a	6.8b	19.8bc	0.011	0.98	<0.001
*Q. ilex*	22.7a	18.4bc	1.4d	33.2b	13.1b	4.1d	6.1ab	11.0a	11.7a	43.1a	7.1ab	17.0c	0.002	0.78	0.022
*Q. pubescens*	23.5a	20.9b	1.9c	25.8c	10.9b	9.7a	6.7ab	12.0a	9.4b	36.0c	7.2ab	19.0bc	0.004	0.94	0.001
Robinia^*^^†^	18.7ab	18.9bc	2.5b	19.7d	7.5c	8.5ab	5.1c	9.2bc	10.3ab	37.0c	8.5a	21.5b	0.008	0.81	0.016
*Ulmus*	19.3a	18.5bc	2.4b	20.6*d*	7.7c	8.7ab	4.6c	8.9c	9.6b	43.2a	6.3b	18.7bc	0.011	0.83	0.013
NC	19.9	16.3	1.6	31.0	10.7	5.2	5.6	10.5	11.1	42.3	7.1	18.2	0.007	0.89	0.009
**MIXED FOREST VINES**															
*Clematis*	10.2*c*	2.4*d*	1.9*b*	25.8*b*	1.2*c*	10.0a	6.6a	14.4a	7.8b	35.9b	6.3b	18.9c	0.008	0.95	0.001
*Hedera*	23.6a	5.7*c*	1.9*b*	26.9b	3.1b	7.2b	2.7b	5.9b	8.9a	43.2a	6.8*b*	25.3a	0.006	0.89	0.004
Ipomea^*^	17.5*b*	11.6a	4.2a	12.0c	2.8b	11.1a	1.0c	5.3b	7.6b	37.0b	10.9a	27.0a	0.013	0.77	0.025
*Rubus*	16.4b	8.1b	1.7b	29.8a	4.8a	6.3b	3.7b	7.9b	8.5a	41.2a	8.6ab	23.7b	0.011	0.98	<0.001
NC	19.4	5.3	1.9	27.1	2.9	7.7	3.8	8.2	8.6	41.2	6.9	23.5	0.008	0.94	0.002

*Values of native communities (NC) are the average of coexisting native species weighted by their relative abundance (Table S2). For each parameter and each community, different letters indicate significantly different groups (Duncan test, P < 0.05). Invasive species are marked by an asterisk, while N-fixing species are marked by*.

All species from the sand dune community showed the opposite trend, with low N content, high lignin content and, consequently, high C/N and lignin/N ratios (Table 2). However, in this community, the invasive tree *Acacia longifolia* showed the highest N content, and a high lignin content, whereas C/N and lignin/N ratios were very low, compared with co-occurring native species (Table 2). Similarly, the invasive species *Eucalyptus camaldulensis* showed a relatively high N content and the lowest lignin/N ratio (Table 2). ^13^C CPMAS NMR data showed that the two invasive species generally had a low relative content of *O*-Alkyl C but a higher fraction of methoxyl and N-alkyl C and alkyl C, compared to native plants (Table 2).

In the mixed forest, all vines obviously showed lower lignin content compared to trees; they also showed low C/N and lignin/N ratios (Table 2). Interestingly, the invasive vine *Ipomea purpurea* showed a very high N content and a low lignin content, leading to very low C/N ratio (Table 2). Considering ^13^C CPMAS NMR data, *I. purpurea* showed high relative content of methoxyl and N-alkyl C and a low fraction of *O*-substituted aromatic C compared to native vines (Table 2). In this community, leaf litter chemistry largely varied among trees species. For instance, C/N and lignin/N ratio ranged from 39.2 (*Arbutus unedo*) to 16.6 (*Ailanthus altissima*) and from 21.0 (*A. unedo*) to 0.8 (*Brussonetia papyrifera*), respectively (Table 2). The invasive tree species basically showed high N content and low C/N ratio species compared to native, with *B. papyrifera* also showing very low lignin content (Table 2). ^13^C CPMAS NMR data showed higher relative content of methoxyl and N-alkyl C in invasive compared to most co-occurring native trees, among which only the N-fixing *A. glutinosa* and *C. australis* showed similarly high values (Table 2). Compared to the dominant native tree (*Quercus ilex*, Table S1), all three woody species invasive of mixed forest community consistently showed lower H-C and O-substituted aromatic fractions, and higher methoxyl and N-alkyl C, carboxylic C and alkyl C (Table 2).

Finally, in the riparian forest, the invasive tree *Amorpha fruticosa* showed the highest N content coupled with the lowest C/N and lignin/N ratio (Table 2). For this species, ^13^C CPMAS NMR analysis revealed a high relative content of carboxylic C and a low fraction of *O*-alkyl C (Table 2).

### Litter mass loss and N dynamics

**Figure 1:**
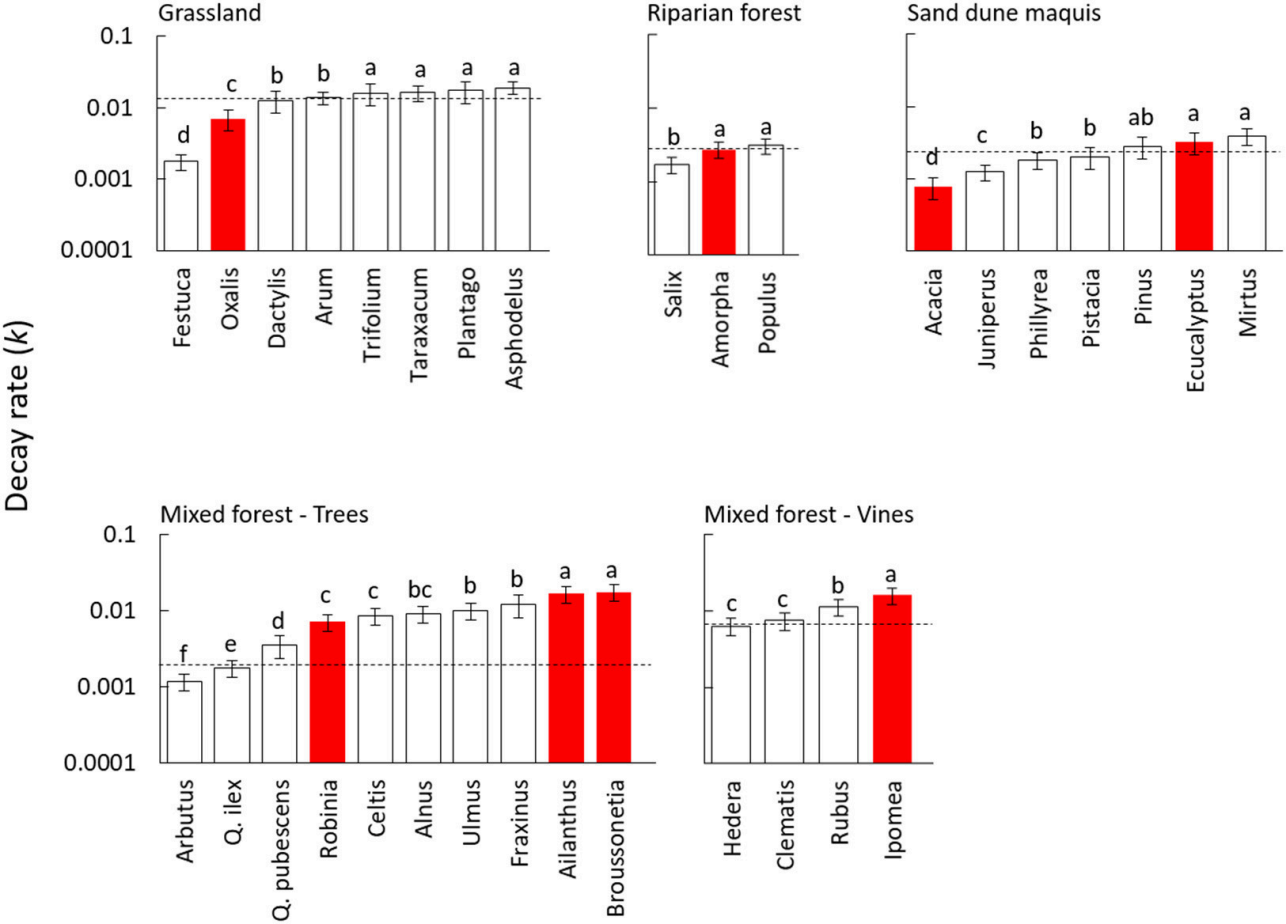
Decay rate (*k*) of leaf litter from 32 plant species decomposed in field conditions for 360 days in different Mediterranean plant communities. White and red bars indicate native and invasive species, respectively. Dashed lines refer to values of the whole native community (NC) calculated as the average of all coexisting species weighed by their relative abundance. Values are average ± standard error, different letters in each panel indicate significantly different groups (Duncan test, *P* < 0.05). The species within each community are ordered by ascending values of decay rate (*k*).

Decomposition dynamics significantly fitted a simple exponential model for all litter species, with decay rates largely varying among different materials (Table 2). Litter mass loss was significantly affected by the plant species source in all tested communities (Table S2), but with differences between invasive and native species widely varying in magnitude and direction across different plant communities. Accordingly, the nativity/invasivity effect in our mixed model for litter decay rate was not significant, whereas effects of site and family were statistically significant (Table 3). In the mixed forest, the invasive trees *A. altissima* and *B. papyrifera* showed the highest decay rate among woody plants, as well as *I. purpurea* did among vines (Figure 1), with values of all invasive species higher than the average of the native community (Figure 1). Differently, in both the grassland and the riparian forest, invasive species showed litter decay rates within the range observed for co-occurring native plants (Figure 1). Finally, in the sand dune, the two invasive plants produced opposite results, with the N-fixing shrub *A. longifolia* and the tree *E. camaldulensis* showing the lowest and the highest decay rates, respectively (Figure 1).

**Table 3:** Summary of Generalized Linear Mixed Modeling (GLMM) results for litter decay rate (*K*) and net nitrogen (N) release, testing fixed effects of plant nativity/invasivity, and random effects of site and phylogenetic groups (i.e., family) and, limited to N release, decomposition time.

	**Effect type**	**d.f**.	**SS**	**MS**	**F**	***P***	**Statistical significance**
**DECAY RATE (*K*)**							
Nativity	Fixed	1	0.000004	0.000004	1.13	0.361	n.s.
Site	Random	3	0.000243	0.000081	15.83	*<0.000*	^***^
Family	Random	17	0.000567	0.000033	14.02	*<0.001*	^***^
Nativity × Site	Random	3	0.000018	0.000006	2.45	0.122	n.s.
**NET N RELEASE**							
Nativity	Fixed	1	845.7	845.7	7.45	*0.007*	^**^
Family	Random	5	17904.5	3580.9	5.21	*0.004*	^**^
Site	Random	3	24461.5	12230.8	11.14	*0.046*	^*^
Time	Fixed	1	1563.2	1563.2	1.60	0.277	n.s.
Nativity × Site	Random	1	558.9	558.9	2.41	0.124	n.s.
Nativity × Time	Fixed	1	31.3	31.3	0.28	0.600	n.s.
Family × Time	Random	19	12760.5	671.6	5.91	*<0.001*	^***^
Site × Time	Random	3	2571.7	857.2	7.55	*<0.001*	^***^

Second order effects are limited to tested effect level combinations. Model coefficients (i.e., estimated slopes and standard error for each effect level) are reported in Table S3. Asterisks indicate statistical significance (

*** P < 0.001;

** P < 0.01;

* *P < 0.05; n.s., not significant)*.

**Figure 2:**
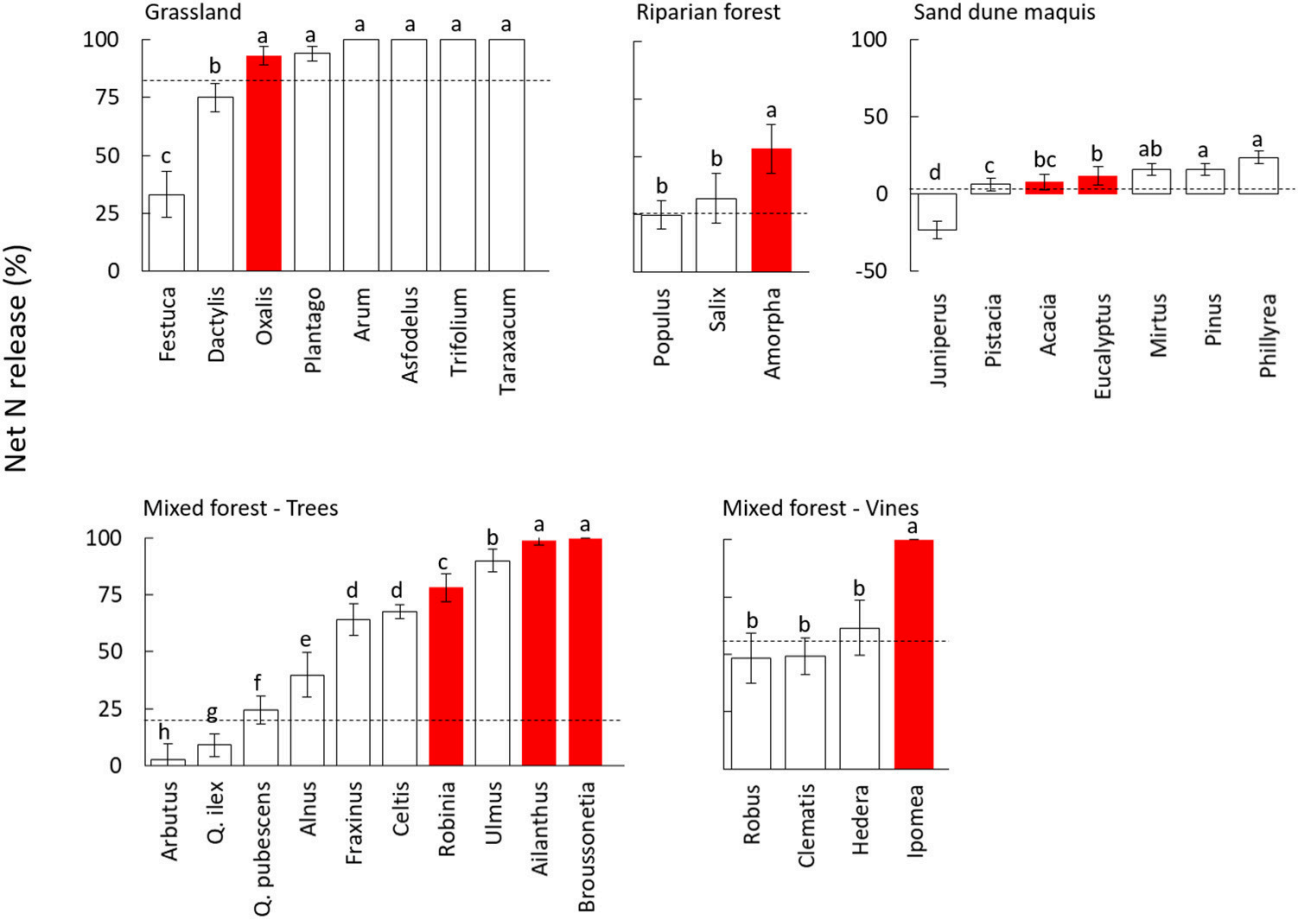
Net nitrogen (N) release (% of N lost with respect to the initial content) after 90 days of decomposition in leaf litter of 32 species from different plant communities. White and red bars indicate native and invasive species, respectively. Dashed lines refer to values of the whole native community (NC) calculated as the average of all coexisting species weighted by their relative abundance. Values are average ± standard error, different letters in each panel indicate significantly different groups (Duncan test, *P* < 0.05). The species within each community are ordered by ascending values of net N release.

A large variability in N transfer was observed after 90 days of decomposition, with net N mineralization observed for most of the species and N immobilization observed only in sand dune ecosystem for the native *J. phoenicea* (Figure 2, Figure S2, and Table S2). Notably, all invasive species showed a rapid N mineralization, with values of net N release higher than the average of the native community in all ecosystems (Figure 2, Figure S2). Accordingly, the nativity/invasivity effect in our mixed model for N release was statistically significant (Table 3). The effects of site and family, and their combined effect with decomposition time were statistically significant as well (Table 3), indicating that the magnitude of net N release significantly varied across different communities and phylogenetic groups, also depending on the decomposition stage. However, the interaction of site and nativity/invasivity was not significant (Table 3), indicating that in all plant communities N mineralization for invasive plants was consistently higher than that of native species. In particular, the trees species *A. altissima* and *B. papyrifera* and the vine *I. purpurea* in the mixed forest, as well as *A. fruticosa* in the riparian forest, showed N release significantly higher than that of all co-occurring native species, while in the grassland community *O. pes-caprae* showed non-significantly different value compared to the observed maximum (Figure 2). Similar results were found after 360 days of decomposition (Figure S2).

### Relationships between litter chemistry, mass loss, and N dynamics

Considering chemical parameters of undecomposed materials, litter decay rate (*k*) was negatively associated to proximate lignin content, and C/N and lignin/N ratios, and positively to N content (Table 4). Among ^13^C CPAMS NMR regions, *k* was negatively correlated with aromatic fractions (Table 4).

**Table 4:** Correlation (Pearson's *r, N* = 32 in all cases) between litter chemical traits and litter mass loss and net nitrogen release (recorded after 90 and 360 days of decomposition).

	**Litter decay rate (*k*)**	**Nitrogen release (% after 90 d)**	**Nitrogen release (% after 360 d)**
**ELEMENTAL AND PROXIMATE PARAMETERS**			
Cellulose (%)	0.04	0.24	0.14
Lignin (%)	−0.66^*^	−0.68^*^	−0.68^*^
N content (%)	0.44^*^	0.59^*^	0.55^*^
C: N ratio	−0.37^*^	−0.59^*^	−0.78^*^
Lignin: N ratio	−0.57^*^	−0.72^*^	−0.92^*^
^13^**C-CPMAS NMR-DERIVED PARAMETERS**			
Alkyl C: 0-45 ppm	−0.02	−0.03	0.03
Methoxyl and N-alkyl C: 46–60 ppm	0.32	0.46^*^	0.47^*^
*O*-alkyl C: 61–90 ppm	0.20	0.25	0.03
di-*O*-alkyl C: 91–110 ppm	−0.17	−0.25	−0.35
H-C-substituted aromatic C: 111–140 ppm	−0.43^*^	−0.62^*^	−0.39^*^
*O*-substituted aromatic C: 141–160 ppm	−0.54^*^	−0.70^*^	−0.50^*^
Carboxylic C: 161–190 ppm	0.28	0.52^*^	0.56^*^

* *Statistically significant correlations (P < 0.01)*.

N dynamics showed a pattern of correlation with litter chemistry parameters very similar to that observed for decay rate, considering net N release after both 90 and 360 days of decomposition. The only differences were the positive association of N release, at both litter ages, with the N-alkyl C and the carboxylic C spectral regions (Table 4).

**Figure 3:**
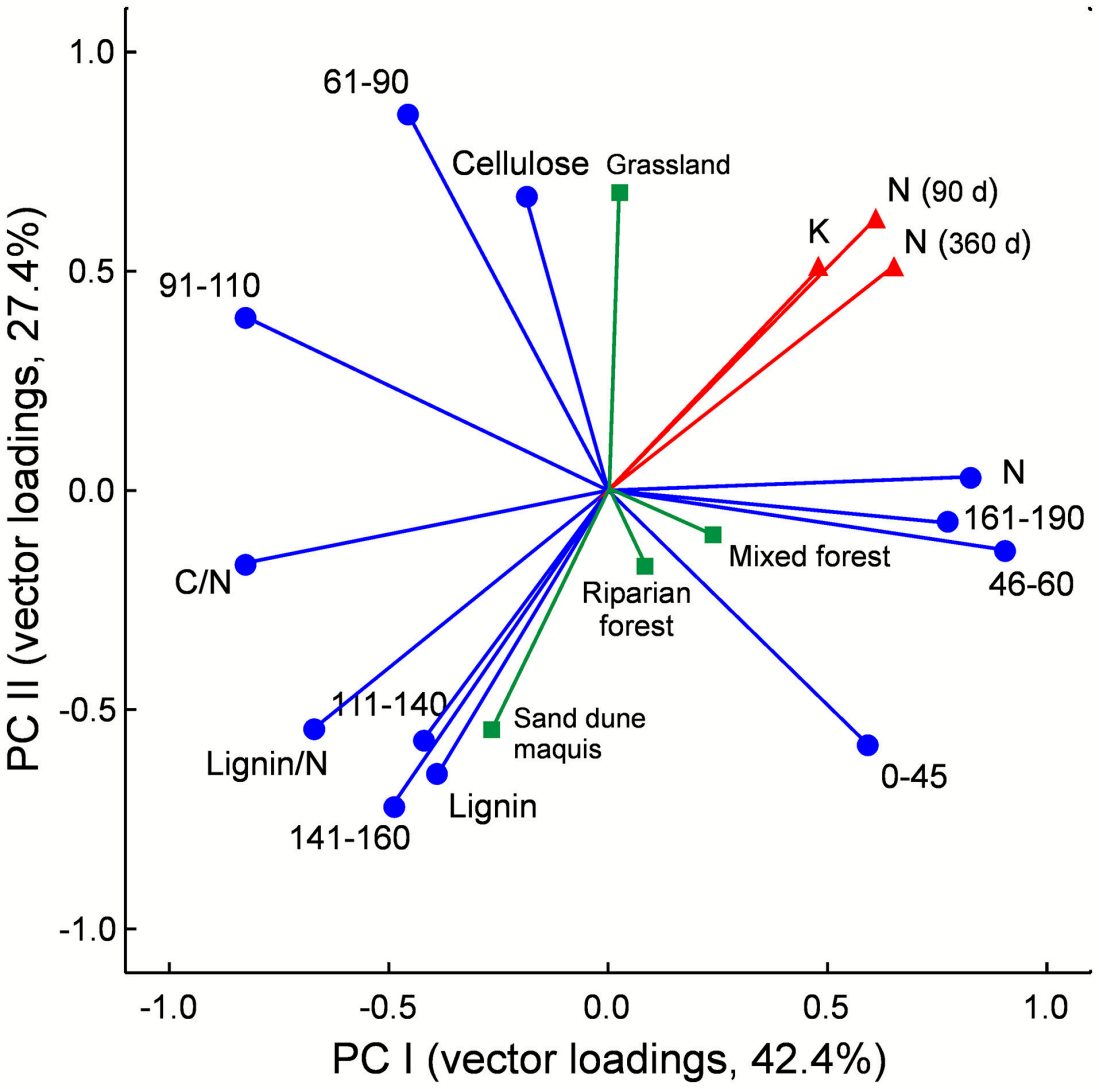
Principal component analysis (PCA) of litter chemical traits (blue vectors), based on values recorded in the 32 litter materials. Data refer to loading vectors of litter chemical parameters assessed by elemental, proximate, and ^13^C-CPMAS NMR spectroscopy analyses. Spectral regions are labeled by resonance interval in ppm, with corresponding C types according to Bonanomi et al. (2013): 0–45 ppm = alkyl C; 46–60 ppm = methoxyl and N-alkyl C; 61–90 ppm = O-alkyl C; 91–110 ppm = di-O-alkyl C; 111–140 ppm = H- and C- substituted aromatic C; 141–160 ppm O-substituted aromatic C (phenolic and O-aryl C); 161–190 ppm carboxyl C. Litter decay rate (*k*), net N release after 90 and 360 days and plant communities are also plotted as supplementary variables (red and green vectors) following Legendre and Legendre (1998).

The principal component analysis (PCA) provided a satisfactory ordination of the litter chemistry parameters, with the first two eigenvalues accounting for 69.81% (42.25 and 27.56%) of the total variance (Figure 3). It synthetically showed the relationships between litter chemistry and both litter decay rate and N mineralization, with the negative association with proximate lignin content, C/N and lignin/N ratios, and the two aromatic C fractions resonating at 111–160 ppm in the ^13^C NMR spectra of the litter materials, the positive correlation with litter N content and the carboxylic C and methoxyl and N-alkyl C fraction, and the minor role of alkyl C, *O*-alkyl C and di-*O*-alkyl C regions, corresponding to lipids and carbohydrates, in explaining C and N dynamics in the litter materials (Figure 3).

## Discussion

### Litter mass loss and N release of native and invasive species

In this study, decomposition rates of leaf residues did not consistently differ among invasive and native plant species. Indeed, litter of invasive plants decomposed at faster rate compared to native species in the mixed forest, when the comparison was limited to either trees or vines growth forms, but the opposite pattern was found in both grassland and sand dune vegetation. In such plant communities, the invasive forb *Oxalis pes-caprae* and the N-fixing shrub *Acacia longifolia* showed lower mass loss rate compared to most co-occurring native plants and also to the weighted average of the native community. Our results contradict early evidence summarized in the reviews by Ehrenfeld (2003) and Liao et al. (2008), where litter decay rate of invasive species was significantly higher than native plants. The meta-analysis by Castro-Díez et al. (2014) reported that the impact of plant invasion on litter decomposition was not significant. In a recent comparative study on 42 native and 36 non-native woody species in temperate deciduous forest, Jo et al. (2016) did not observe systematic differences between the two groups. The authors argued that the common view behind fast decomposition of invasive plant litter is based on the systematic bias intrinsic to the plant species selection in previous experimental studies, with inclusion of invader species with large impact on ecosystem functions while disregarding the whole native plant community. In this context, our study including all invasive species and most of the native plants in each ecosystem type is certainly not affected by biased selection. Moreover, our results support the finding by Jo et al. (2016) since some invaders showed exceptionally high decay rate but, overall, litter mass loss was not systematically different between invasive and native plants.

One of the most remarkable results of our study was that invasive litter released N at faster rate compared with native species, independent of the considered plant community. Such difference certainly varied in different plant communities, with mean values of 13, 79, 107, 323, and over 400% for invasive herbs in grassland, vines in mixed forest, and woody trees in riparian forest, mixed forest, and sand dunes, respectively. Consistently, N release from each invasive species was faster than the average of the invaded community. In general terms, our results about N release are in agreement with previous observations referring to other ecosystems, as summarized in the meta-analysis of Liao et al. (2008) and Castro-Díez et al. (2014). As a novel point, we extend such findings to so far unexplored Mediterranean ecosystems.

### Linking litter chemical traits, mass loss, and N release

Litter chemical traits significantly varied between invasive and native species. In general, invaders had higher N content and lower lignin/N ratio compared to coexisting native species, with the only exception of *Oxalis pes-caprae* in the grassland ecosystem. Moreover, the methoxyl and N-alkyl C fraction assessed by ^13^C CPMAS NMR, which relates to proteins and peptides but also to lignin components, was basically higher in invasive plants, especially considering N-fixing woody species.

Simple regression and PCA analyses provided indication of the chemical traits likely controlling litter mass loss and N mineralization, i.e., N concentration and proximate lignin content, lignin/N ratio and the methoxyl and N-alkyl C NMR regions. In detail, rates of C and N mineralization were negatively associated to proximate lignin content and positively to N concentration. This finding, indeed, is not new, but confirmative of well-established previous knowledge (Meentemeyer, 1978; Melillo et al, 1982; Taylor et al., 1989; Bonanomi et al., 2013). However, at this stage, a key question arises from our results: if litter mass loss and N mineralization are controlled by the same chemical traits, why invasive species had similar decay rate compared to co-occurring native plants, but a faster N mineralization? The extensive characterization of litter chemical traits carried out in this study, combining element and proximate analyses with ^13^C CPMAS NMR data, could help to shed light on this question.

Proximate lignin content was negatively associated to both decay rate and N mineralization, with very similar correlation scores. Differently, litter N content showed a higher coefficient of correlation with N mineralization than with mass loss, indicating a stronger relationship. Then, it could be argued that, at similar levels of lignin content, litter materials with lower lignin/N ratio because of higher N content, as we observed for invasive species, promote N mineralization more than mass loss. Castro-Díez et al. (2014) reported that invasive species, in spite of a lower C/N ratio, have similar decay rate compared to co-occurring native plants. Accordingly, Jo et al. (2016) recently found higher N content in invasive than in native plant litter, but mass loss rates were not significantly different. The authors suggested that a high lignin content in invasive plant litter constrains mass loss, thus obscuring the enhancing effect of the high N content. Our results support this hypothesis limited to mass loss dynamics, but not for N mineralization that, in our study case, is strictly controlled by the initial litter N content. Results of litter analysis by ^13^C CPMAS NMR further supports this hypothesis.

The relative abundance of the methoxyl and N-alkyl C region was positively correlated with N mineralization but not with litter mass loss. Such NMR spectral region can be associated in litter to aminoacidic alpha-carbon, hence to peptides and protein content, whereas methoxyl C are indicative of lignin content (Kögel-Knabner, 2002). Since the spectral signals of N-alkyl and methoxyl carbons overlap in the spectral region at 46–60 ppm, a high peak in this region alone does not conclusively clarify whether the sample has a high content of N and/or a high content of lignin. Interestingly, litter materials from the invasive, N-fixing woody species included in this study (i.e., *Acacia longifolia, Amorpha fruticosa*, and *Robinia pseudoacacia*, Table S1) all showed high peaks in this spectral region, which corresponded to high contents of N, recorded by elemental analyses, and proximate lignin. These leaf materials consistently underwent fast processes of N mineralization, not observed for mass loss, as related to such peculiar litter chemistry. Clearly, this result suggests that the initial N content of litter promotes N mineralization more than C loss. In this regard, it is also well known that the initial N content has a dual effect on litter decomposition depending on its stage; while it enhances mass loss during the first decay stages (up to 30–40% of mass loss) it has a limiting effect in later stages (Berg and Matzner, 1997). During the early phases of the process, a high N content can sustain large microbial populations that rapidly consume labile C compounds which results in a faster mass loss. Inversely, a high N concentration inhibits mass loss at later stages by favoring the formation of recalcitrant chemical complexes with lignin, even though the microbiological and biochemical mechanisms have not yet been fully explained (Hatakka, 2001; Berg and McClaugherty, 2014).

### Implications at plant community scale

Invader species are expected to affect N cycle in all the Mediterranean plant communities considered in our study. In mixed forest, the three invasive species showed faster C and N loss compared with native plants. The impacts of *Ailanthus altissima* and *Robinia pseudoacacia* on Mediterranean ecosystems have been widely investigated (Vilà et al., 2006; González-Muñoz et al., 2013), while no studies were specifically focused on *Broussonetia papyrifera*. Our data, being not affected by species-selection bias as based on the whole-community approach (Hulme et al., 2013), indicate that C and N mineralization rates of invasive plants are strikingly high especially when compared to the most abundant native tree, *Quercus ilex*, an evergreen sclerophyll oak with high lignin and very low N leaf content. As a consequence, a large impact can be expected on C and N stocks and fluxes in Mediterranean evergreen and mixed forests due to this multiple species invasion (Kuebbing et al., 2013). Such impact could also be exacerbated by the invasive vines such as *Ipomea purpurea*. Despite all native and invasive vines underwent fast C and N mineralization, *I. purpurea* showed the maximum rates, releasing 100% of the initial N content after 90 days of decomposition, as compared to 38, 51, and 52% of the native vines *Hedera helix, Clematis vitalba*, and *Rubus ulmifolius* over the same period. As discussed above, the combination of high N content, high methoxyl and N-alkyl C fraction, and low proximate lignin content, could explain the response of *I. purpurea* litter to microbial decomposition.

*Amorpha fruticosa* has been reported as an aggressive invader of riparian plant communities worldwide, including Japan (Takagi and Hioki, 2013), US (Glad and Halse, 1993), and Europe (Quézel et al., 1990). However, no previous studies addressed its impact on the N cycle of invaded ecosystems. Litter from such N-fixing tree underwent faster N mineralization compared to the native *Pinus nigra* and *Salix alba*, presumably due to high leaf N content and low lignin/N ratio. Accordingly, its impact on C and N cycle in Mediterranean riparian forests is expected to be high, also because of the absence of native N-fixing trees or shrubs that may competitively exclude *A. fruticosa* from the invaded system.

Marchante et al. (2008) studied the long-term impact of *Acacia longifolia* on Mediterranean sand dunes, reporting an increase of soil organic C stock 20 years after the invasion, ascribed to high litter fall. Consistently, our results indicate that chemical traits of *A. longifolia* litter, such as a relatively high N concentration combined with very high proximate lignin content, could slow down litter C loss, while counterintuitively enhancing N mineralization rate. The combined effects of such processes may consist in an increase of soil organic C stock coupled with N depletion, with potential positive feedback on further invasion. On the other hand, the impact of *Eucalyptus camaldulensis* invasion on Mediterranean sand dunes has not yet been investigated. This evergreen tree showed litter mass loss and N release slightly higher than the average of the invaded community, apparently unaffected by the extremely high ^13^C NMR alkyl C fraction, which typically indicates the content of lipids such as waxes, cutins, and oils (Kögel-Knabner, 2002). However, the impacts of significant inputs of such chemical compounds by *E. camaldulensis* invasion on soil quality, microbial and native plant communities are unknown, and certainly deserve further investigation.

Finally, the grassland invasion by *Oxalis pes-caprae*, which is recognized as one of the most important invasive species in the Mediterranean biome (Gimeno et al., 2006) likely had the most limited impact, among the tested cases, on local C and N cycles. Indeed, *O. pes-caprae* was the only invasive plant to show lower litter N content and higher lignin/N ratio compared to most of the co-occurring native species. However, its litter N release was slightly faster than the average of the native community. Then, considering that this species has earlier phenology compared to co-occurring herbs, it can be expected that invaded grasslands show significant N release to the soil in early spring, whose possibly detrimental effect on native plant community, or a shift toward more nitrohytic assemblages, should be effectively assessed.

## Conclusions

Our field assessment of litter mass loss for 32 species in 4 different plant communities revealed no consistent differences between native and invasive species, although some woody and vine invaders showed exceptionally high decomposition rate. In contrast, N release was faster during litter decomposition of the invasive plants compared to native species in all the tested communities. N concentration, proximate lignin content and the fraction of methoxyl and N-alkyl C from ^13^C CPMAS NMR spectra, linked to aminoacid and lignin content, were the chemical parameters that better explained mass loss and N mineralization. A significant difference in such chemical traits was found between native and invasive species. According to our findings, invader species are expected to affect N cycle in Mediterranean plant communities, possibly promoting a shift toward different plant assemblages and further invaision of exotic species.

## Author contributions

GI, FC, GB, and FG conceived the study, designed the work and conducted the data analysis. GB, AA, TS, GC, and RD collected litter samples, carried out the decomposition experiment and made chemical analyses. GB, AA, and RD contributed to data analyses. GI and FC wrote the manuscript, with contributions from all the other authors, FG revised the final draft.

### Conflict of interest statement

The authors declare that the research was conducted in the absence of any commercial or financial relationships that could be construed as a potential conflict of interest.
