# Supplementary material for: Faster N Release, but Not C Loss, From Leaf Litter of Invasives Compared to Native Species in Mediterranean Ecosystems
Source: Front Plant Sci. 2018 Apr 24;9:534. doi: 10.3389/fpls.2018.00534 (PMC5928551; doi:10.3389/fpls.2018.00534)
Supplement: Supplementary file 1 [file Table_1.pdf]

frontiers

Supplementary Material

Faster N Release, but Not C Loss, from Leaf Litter of Invasives Compared to Native Species in Mediterranean Ecosystems

Guido Incerti[1,§], Fabrizio Cartenì[2,§], Gaspare Cesarano[2], Tushar C. Sarker[2], Ahmed M. Abd El-Gawad[3], Rosaria D'Ascoli[4], Giuliano Bonanomi[2], Francesco Giannino[2,*]

*** Correspondence:**
dr. Francesco Giannino
E-mail: giannino@unina.it

1 Supplementary Tables

Supplementary Table S1. Species composition and relative abundance, expressed as percent cover, in the four plant communities. Asterisks indicate invasive species.

Ecosystem	Species	Cover (%)	Growth form	Status
Grassland	*Dactylis*	44	Perennial grass	Native
	Asphodelus	13	Perennial forb	Native
	Taraxacum	12	Perennial forb	Native
	Festuca	9	Perennial grass	Native
	Plantago	8	Perennial forb	Native
	Trifolium	8	Perennial forb – N-fixing	Native
	Arum	5	Perennial forb	Native
	*Oxalis**	11	Perennial forb	Invasive
Riparian forest	*Populus*	75	Deciduous tree	Native
	Salix	12	Deciduous tree	Native
	*Amorpha**	14	Deciduous tree – N-fixing	Invasive
Sand dune	*Pistacia*	21	Evergreen shrub	Native
	Juniperus	18	Evergreen shrub	Native
	Mirtus	17	Evergreen shrub	Native
	Pinus	12	Evergreen tree	Native
	Phillyrea	9	Evergreen shrub	Native
	*Acacia**	18	Evergreen shrub – N-fixing	Invasive
	*Eucalyptus**	7	Evergreen tree	Invasive
Mixed forest	*Q. ilex*	58	Evergreen tree	Native
	Fraxinus	7	Deciduous tree	Native
	Arbutus	5	Evergreen tree	Native
	Celtis	5	Deciduous tree	Native
	Olmus	3	Deciduous tree	Native
	Alnus	3	Deciduous tree – N-fixing	Native

	Q. pubescens	3	Deciduous tree	Native
	*Ailanthus**	5	Deciduous tree	Invasive
	*Brussonetia**	7	Deciduous tree	Invasive
	*Robinia**	9	Deciduous tree – N-fixing	Invasive
	Hedera	13	Vine	Native
	Clematis	4	Vine	Native
	Rubus	3	Vine	Native
	*Ipomea**	5	Vine	Invasive
