# Supplementary material for: Faster N Release, but Not C Loss, From Leaf Litter of Invasives Compared to Native Species in Mediterranean Ecosystems
Source: Front Plant Sci. 2018 Apr 24;9:534. doi: 10.3389/fpls.2018.00534 (PMC5928551; doi:10.3389/fpls.2018.00534)
Supplement: Supplementary file 2 [file Table_2.pdf]

Supplementary Material

Faster N Release, but Not C Loss, from Leaf Litter of Invasives Compared to Native Species in Mediterranean Ecosystems

Guido Incerti[1,§], Fabrizio Cartenì[2,§], Gaspare Cesarano[2], Tushar C. Sarker[2], Ahmed M. Abd El-Gawad[3], Rosaria D'Ascoli[4], Giuliano Bonanomi[2], Francesco Giannino[2,*]

*** Correspondence:**
dr. Francesco Giannino
E-mail: giannino@unina.it

1 Supplementary Tables

Supplementary Table S2. Summary of two-way ANOVA results for litter mass loss and nitrogen (N) content in each plant community, testing main and interactive effects of litter type and decomposition time. Asterisks indicate statistical significance (**, *P* < 0.01; *, *P* < 0.05; n.s., not significant).

	d.f.	SS	MS	F	*P*	Statistical significance
Mass loss (tree in mixed forest)						
Species (S)	9	46446	5160	1853	< 0,01	**
Decomposition time (T)	4	142027	35506	12753	< 0,01	**
S x T	36	16954	470	169	< 0,01	**
Mass loss (riparian forest)						
Species (S)	2	992	496	299	< 0,01	**
Decomposition time (T)	4	25260	6315	3816	< 0,01	**
S x T	8	442	55	33	< 0,01	**
Mass loss (sand dune)						
Species (S)	5	6127	1225	1202	< 0,01	**
Decomposition time (T)	4	34666	8666	8504	< 0,01	**
S x T	20	3793	189	186	< 0,01	**
Mass loss (vine in mixed forest)						

Species (S)	3	4604	1534	448	< 0,01	**
Decomposition time (T)	4	72283	18820	5494	< 0,01	**
S x T	12	4681	390	113	< 0,01	**
Mass loss (grassland)						
Species (S)	6	20215	3369	891	< 0,01	**
Decomposition time (T)	4	141910	35477	9391	< 0,01	**
S x T	24	9933	413	109	< 0,01	**
Nitrogen content (tree in mixed forest)						
Species (S)	9	61194	6799	728	< 0,01	**
Decomposition time (T)	1	5967	5967	639	< 0,01	**
S x T	9	5010	556	59	< 0,01	**
Nitrogen content (riparian forest)						
Species (S)	2	2231	1115	252	< 0,01	**
Decomposition time (T)	1	2919	2919	661	< 0,01	**
S x T	2	274	137	31	< 0,01	**
Nitrogen content (sand dune)						
Species (S)	6	13491	2248	856	< 0,01	**
Decomposition time (T)	1	6634	6634	2526	< 0,01	**
S x T	6	2049	341	130	< 0,01	**
Nitrogen content (vines in mixed forest)						
Species (S)	3	3327	5374	101	< 0,01	**
Decomposition time (T)	1	5374	5374	493	< 0,01	**
S x T	3	2473	824	75	< 0,01	**
Nitrogen content (grassland)						
Species (S)	6	28461	4743	354	< 0,01	**
Decomposition time (T)	1	39	39	2	0,09	n.s.
S x T	6	567	94	7	< 0,01	**
