# Supplementary material for: Faster N Release, but Not C Loss, From Leaf Litter of Invasives Compared to Native Species in Mediterranean Ecosystems
Source: Front Plant Sci. 2018 Apr 24;9:534. doi: 10.3389/fpls.2018.00534 (PMC5928551; doi:10.3389/fpls.2018.00534)
Supplement: Supplementary file 3 [file Table_3.pdf]

# *Supplementary Material*

# Faster N Release, but Not C Loss, from Leaf Litter of Invasives Compared to Native Species in Mediterranean Ecosystems

Guido Incerti[1,§], Fabrizio Carteni̇̀[2,§], Gaspare Cesarano[2], Tushar C. Sarker[2], Ahmed M. Abd El-Gawad[3], Rosaria D'Ascoli[4], Giuliano Bonanomi[2], Francesco Giannino[2,*]

*** Correspondence:**
dr. Francesco Giannino
E-mail: giannino@unina.it

## 1 Supplementary Tables

**Supplementary Table S3**. Coefficients for each effect level tested in Generalized Linear Mixed Models fitted for litter decay rate (*K*) and net nitrogen (N) release (results in Table 3 in main text). Models included fixed effect of plant nativity/invasivity, and random effects of site and phylogenetic groups (i.e. family) and, limited to N release, decomposition time. For each effect level, data refer to standardized estimate of slope (Beta) and associated standard error, and *P*-value of one-sample *t*-test for significant difference from 0. Signifcant *P*-values are reported in italic font. For each effect, the baseline level is indicated with Beta=0.

| | | **Decay rate (*K*)** | | | **N release** | | |
|---|---|---|---|---|---|---|---|
| **Effect and level** | **Type** | **Beta (ß)** | **S.E. ß** | ***P*** | **Beta (ß)** | **S.E. ß** | ***P*** |
| Nativity | | | | | | | |
| Invasive | Fixed | -0.317 | 0.204 | 0.125 | 0.149 | 0.056 | *0.022* |
| Native | Fixed | 0 | | | 0 | | |
| Family | | | | | | | |
| *Fabaceae* | Random | 0.043 | 0.156 | 0.695 | 0.000 | 0.073 | 1.000 |
| *Simaroubaceae* | Random | 0.214 | 0.095 | *0.017* | -0.007 | 0.054 | 0.901 |
| *Ericaceae* | Random | -0.367 | 0.047 | < *0.001* | -0.505 | 0.066 | < *0.001* |
| *Liliaceae* | Random | 0.019 | 0.095 | 0.692 | 0.000 | 0.039 | 1.000 |
| *Moraceae* | Random | 0.215 | 0.095 | *0.016* | -0.007 | 0.054 | 0.901 |
| *Cannabaceae* | Random | -0.083 | 0.047 | 0.181 | -0.085 | 0.066 | 0.199 |
| *Ranunculaceae* | Random | -0.202 | 0.095 | 0.057 | -0.026 | 0.039 | 0.502 |
| *Poaceae* | Random | -0.297 | 0.128 | *0.032* | -0.281 | 0.046 | < *0.001* |
| *Araliaceae* | Random | -0.244 | 0.095 | *0.020* | -0.069 | 0.039 | 0.075 |
| *Myrtaceae* | Random | 0.153 | 0.093 | 0.069 | 0.109 | 0.084 | 0.194 |
| *Oleaceae* | Random | 0.034 | 0.066 | 0.426 | -0.149 | 0.089 | 0.097 |
| *Cupressaceae* | Random | 0.004 | 0.067 | 0.740 | -0.523 | 0.071 | < *0.001* |
| *Convolvulaceae* | Random | 0.140 | 0.164 | 0.338 | 0.060 | 0.055 | 0.272 |

| | | | | | | | |
|---|---|---|---|---|---|---|---|
| *Fagaceae* | Random | -0.424 | 0.057 | < *0.001* | -0.403 | 0.088 | < *0.001* |
| *Anacardiaceae* | Random | 0.027 | 0.067 | 0.494 | -0.116 | 0.071 | 0.107 |
| *Ulmaceae* | Random | -0.019 | 0.095 | 0.845 | -0.077 | 0.066 | 0.245 |
| *Betulaceae* | Random | -0.074 | 0.047 | 0.245 | -0.083 | 0.066 | 0.210 |
| *Oxalidaceae* | Random | -0.030 | 0.164 | 0.944 | 0.043 | 0.055 | 0.430 |
| *Pinaceae* | Random | 0.070 | 0.067 | 0.190 | -0.020 | 0.071 | 0.777 |
| *Salicaceae* | Random | -0.163 | 0.087 | 0.102 | -0.219 | 0.089 | *0.015* |
| *Plantaginaceae* | Random | 0.016 | 0.095 | 0.718 | 0.000 | 0.039 | 1.000 |
| *Rosaceae* | Random | -0.075 | 0.095 | 0.557 | 0.000 | 0.039 | 1.000 |
| *Asteraceae* | Random | 0.010 | 0.095 | 0.763 | 0.000 | 0.039 | 1.000 |
| *Araceae* | Random | 0 | | | 0 | | |
| Site | | | | | | | |
| Sand dune maquis | Random | -1.030 | 0.159 | < *0.001* | 0.609 | 0.143 | < *0.001* |
| Mixed forest | Random | -0.189 | 0.219 | 0.391 | -0.178 | 0.143 | 0.216 |
| Riparian forest | Random | -0.417 | 0.159 | *0.011* | 0.070 | 0.091 | 0.444 |
| Grassland | Random | 0 | | | 0 | | |
| Time | | Not included in the model | | | | | |
| 90 | Fixed | | | | 0.000 | 0.111 | 1.000 |
| 360 | Fixed | | | | 0 | | |
