# Supplementary material for: Faster N Release, but Not C Loss, From Leaf Litter of Invasives Compared to Native Species in Mediterranean Ecosystems
Source: Front Plant Sci. 2018 Apr 24;9:534. doi: 10.3389/fpls.2018.00534 (PMC5928551; doi:10.3389/fpls.2018.00534)

Figure S1. Air temperature and rainfall time series recorded from the meteorological stations closest to the study sites during the decomposition period (from September 2012 to September 2013). Mean annual temperature and total rainfall were 17.3°C and 1,195 mm for Portici, 17.5 °C and 1,491 mm for Paestum, and 16.9°C and 1,328 mm for Cicerale. Distance from the meteorological stations to the study sites were 4, 8, and 9 km for Portici, Paestum and Cicerale, respectively.

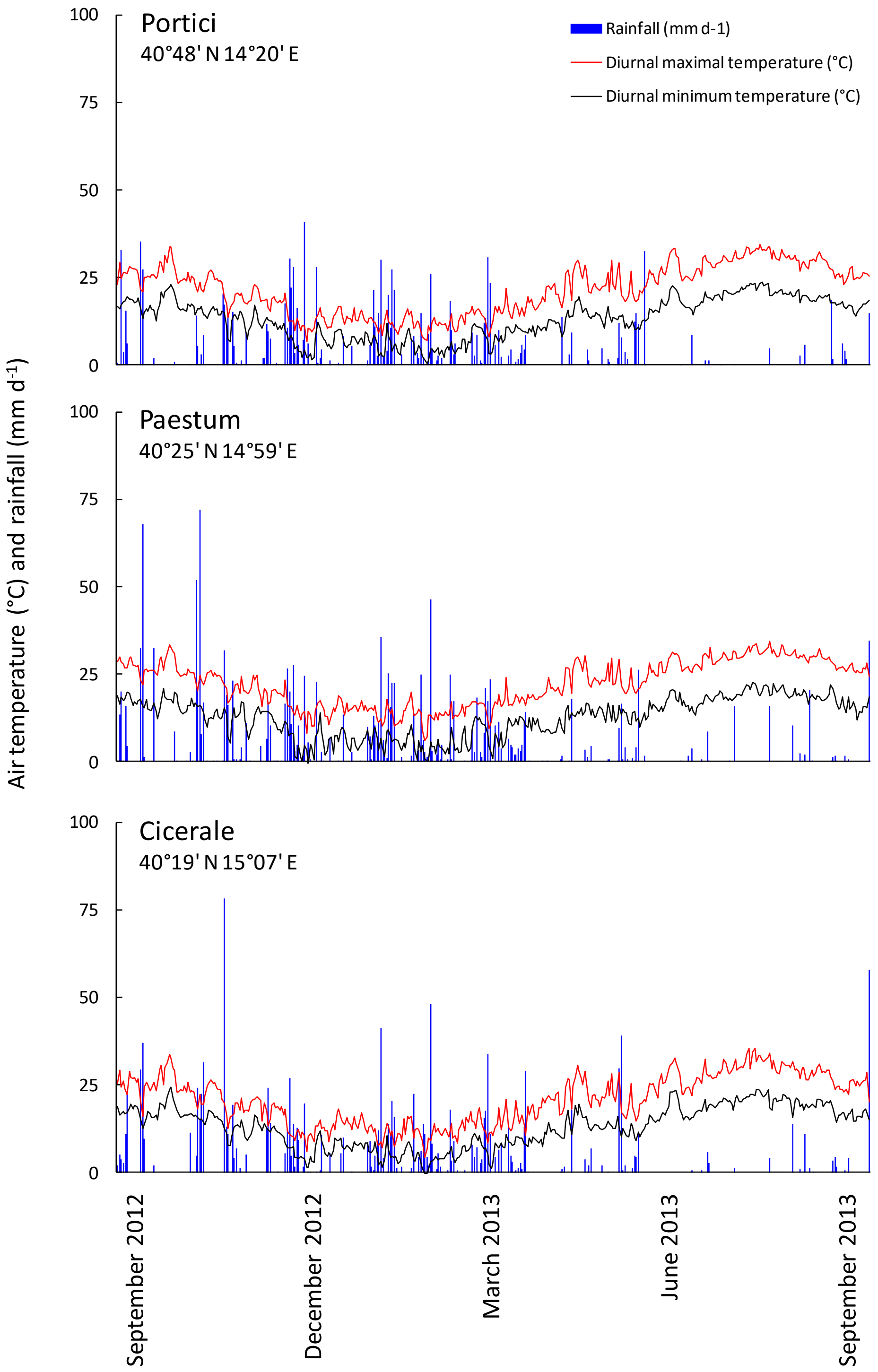

Supplement: Supplementary file 4 [file Image_1.PDF]
