# Supplementary material for: Faster N Release, but Not C Loss, From Leaf Litter of Invasives Compared to Native Species in Mediterranean Ecosystems
Source: Front Plant Sci. 2018 Apr 24;9:534. doi: 10.3389/fpls.2018.00534 (PMC5928551; doi:10.3389/fpls.2018.00534)

Figure S2. Net nitrogen (N) release (% of N lost with respect to the initial content) after 360 days of decomposition in leaf litter of 32 species from different plant communities. Grey and red bars indicate native and invasive species, respectively. Black bars and dashed lines refer to values of the whole native community (NC) calculated as the average of all coexisting species weighted by their relative abundance. Values are average ± standard error, different letters in each panel indicate significantly different groups (Duncan test, $P < 0.05$).

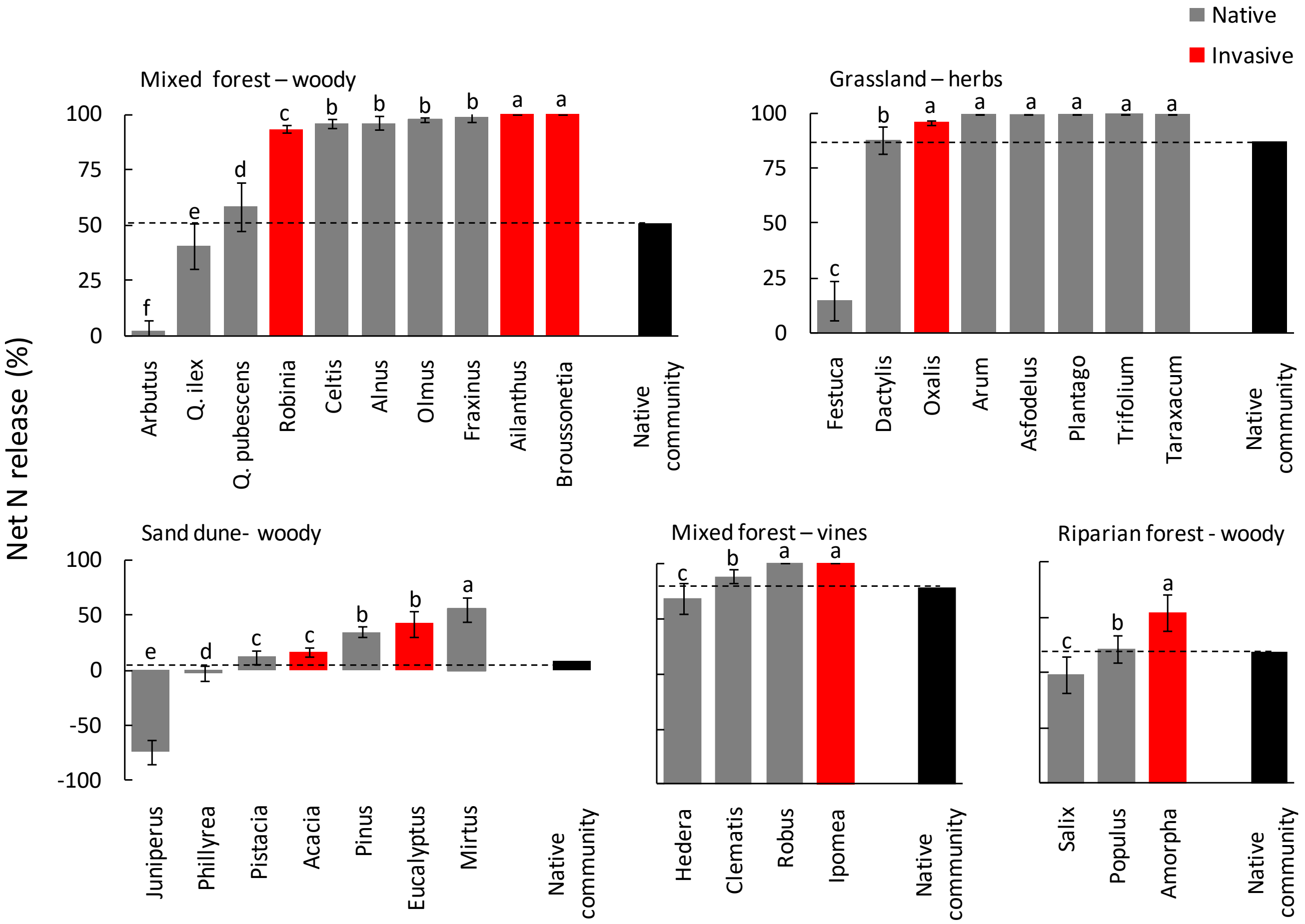

Supplement: Supplementary file 5 [file Image_2.PDF]
